# Analysis of TaqMan Array Cards Data by an Assumption-Free Improvement of the *maxRatio* Algorithm Is More Accurate than the Cycle-Threshold Method

**DOI:** 10.1371/journal.pone.0165282

**Published:** 2016-11-09

**Authors:** Luigi Marongiu, Eric Shain, Lydia Drumright, Reidun Lillestøl, Donald Somasunderam, Martin D. Curran

**Affiliations:** 1 Department of Medicine, University of Cambridge, Cambridge, Cambridgeshire, CB2 0QQ, United Kingdom; 2 Grove Street Technology LLC, 459 Grove Street, Glencoe, Illinois, 60022, United States of America; 3 Public Health England, Clinical Microbiology and Public Health Laboratory, Addenbrooke's Hospital, Hills Road, Cambridge, Cambridgeshire, CB2 0QW, United Kingdom; University of Helsinki, FINLAND

## Abstract

Quantitative PCR diagnostic platforms are moving towards increased sample throughput, with instruments capable of carrying out thousands of reactions at once already in use. The need for a computational tool to reliably assist in the validation of the results is therefore compelling. In the present study, 328 residual clinical samples provided by the Public Health England at Addenbrooke's Hospital (Cambridge, UK) were processed by TaqMan Array Card assay, generating 15 744 reactions from 54 targets. The amplification data were analysed by the conventional cycle-threshold (CT) method and an improvement of the *maxRatio* (MR) algorithm developed to filter out the reactions with irregular amplification profiles. The reactions were also independently validated by three raters and a consensus was generated from their classification. The inter-rater agreement by Fleiss' kappa was 0.885; the agreement between either CT or MR with the raters gave Fleiss' kappa 0.884 and 0.902, respectively. Based on the consensus classification, the CT and MR methods achieved an assay accuracy of 0.979 and 0.987, respectively. These results suggested that the assumption-free MR algorithm was more reliable than the CT method, with clear advantages for the diagnostic settings.

## Introduction

Quantitative polymerase chain reaction (qPCR) has become the standard assay for many diagnostic and research procedures. The throughput of the qPCR has risen from few to hundreds of reactions per run; qPCR plates allocating thousands of reactions are already available. The operators are therefore faced with an increased labour burden detrimental to other laboratory duties and that rises the risk of issuing wrong results.

Analysis of qPCR data relies on a series of fluorescence measurements generated periodically during the reaction of amplification that are interpreted by a variety of algorithms, the most commonly used being the cycle-threshold (CT) method. This approach considers a given threshold of fluorescence and identifies the fractional cycle where the signal crosses the threshold as the quantitative cycle (Cq), also known as threshold (Ct) or crossing-over (Cp) cycle [1,2]. One way of assigning the threshold is to consider the fluorescence generated by the baseline range (cycles 3–15 or 5–9 according to the thermal-cycler system) and to define the cut-off level at tenfold the standard deviation of the fluorescence generated within these cycles [3,4] More commonly, the threshold is chosen subjectively by the operator with the general caveat that it has to be placed in the exponential phase of the amplification [5], therefore the threshold value is often the result of an assumption. In addition, the CT method presumes that the efficiency of amplification is optimal throughout the course of the reaction and equivalent between samples [6]. A plethora of analytical methods have been developed to overcome these assumptions, for instance second derivative maximum [7], sigmoid curve fitting [8–12] and linear regression [13,14].

More recently, the *maxRatio* (MR) method has been introduced [15]. This algorithm produces, for each reaction, both a fractional cycle number (FCN), equivalent to the Cq, and a relative measure of reaction efficiency (MR). The distribution of these values in the FCN-MR plane can be used to discriminate between positive and negative samples in the absence of assumptions from the operator [16]. The MR method is currently included in the Abbott Molecular*m2000 RealTime System* to identify reactive results.

The TaqMan Array Cards (TAC) assay is a contemporary development for qPCR analysis that allows for the simultaneous detection of up to 384 targets using monoplex hydrolysis-probes [17,18]. TAC assays have been developed to detect several microbial pathogens, to monitor the immune response and to assess gene expression alterations in cancer [19–22]. TAC is typically processed on *Viia/QuantStudio* platforms (ThermoFisher Scientific) that only implement the CT method.

The purpose of the present study was to assess whether the MR algorithm could be applied to the analysis of TAC data generated from clinical samples. The present study also sought to improve the original MR algorithm by introducing a filtering protocol to reject reactions on the base of their localisation in the FCN-MR space. The results obtained herein hinted to an increased accuracy of the MR algorithm over the CT method. The application of the MR method as a computer-assisted implement could be beneficial for high throughput qPCR analysis in the clinical and research settings.

## Materials and Methods

### Clinical samples

The clinical samples were obtained between March and May 2015 from residual faecal specimens processed by the Public Health England (PHE) Clinical Microbiology and Public Health Laboratory at Addenbrooke's Hospital (Cambridge, UK). The samples were anonymised prior to analysis.

### Nucleic acid extraction

The faecal samples underwent a bead-beating pre-treatment carried out by adding faecal material to a solution of 900 μl of L6 lysis buffer (ThermoFisher Scientific) and 30 μl of isoamyl alcohol (Sigma-Aldrich). The suspension was placed in a *MagNa Lyser* tube (Roche) and shaken for 1 minute at 3000 rotations per minute in a *MagNa Lyser* vortex (Roche). After shredding, 300 μl of supernatant were mixed to 300 μl of PBS, vortexed, spun down and inserted into a *QIAsymphony SP* automatic extractor (QIAgen). During the extraction process, the samples were spiked with 10^2^ plaque forming units of Bacteriophage MS2 and 10^5^ colony forming units of *Bacillus thuringiensis* in AVE buffer (QIAgen) as external controls of extraction. The nucleic acids were purified using the *DSP virus/pathogen mini* kit (QIAgen), eluted in 110 μl of AVE buffer (QIAgen) and stored at –20°C until TAC analysis.

### TAC assay

The TAC reaction contained 58 μl of nuclease free water, 26 μl of *TaqMan Fast Virus 1-Step Master Mix 2×* (ThermoFisher Scientific) with ROX passive reference dye and 20 μl of nucleic acid extract; 100 μl of such suspension were transferred to a *Custom TaqMan Array 384-well Card* (ThermoFisher Scientific). Lyophilized primers and probes were spotted on the TAC plate's wells during the manufacturing phase; the probes were modified with 6-carboxyfluorescein (FAM) at the 5' end as reporter dye and with NFQ-MGB at the 3' end as quencher. The TAC cards were spun twice at 1200 rpm for 2 minutes, sealed and placed into a *Viia7* thermal cycler (ThermoFisher Scientific). The amplification conditions were: 50°C for 5 minutes for the reverse transcription (RT) step, 95°C for 20 seconds for the initial DNA melting and inactivation of RT reaction, followed by 45 cycles of 95°C for 1 second and 60°C for 20 seconds. The FAM fluorescence was detected on blue filter (520 nm) whereas the ROX emission was recorded on orange filter (610 nm); the fluorescence output was obtained by dividing the emission at 520 nm by that at 610 nm.

### Data acquisition

The amplification data were analysed by the CT method using the *Viia7* version 1.2 software (ThermoFisher Scientific) with a threshold value of 0.2 fluorescence units and a baseline range set to automatic assignment; by default this range was considered between cycles 3 and 15, but it was adjusted according to the actual fluorescence produced in a given reaction. The amplification data were transformed into flat file format (.txt) and used for subsequent analysis and manipulation within the R environment [23].

### Data classification

Three trained operators, referred herein as raters, independently assessed the available data by visually inspecting the amplification profile of each reaction; the raters then issued the reaction as either positive or negative. A consensus classification was established by calculating the median value of the classification of the raters for each reaction. No multicomponent or raw data plots were used for the classification.

### Data resampling

A training dataset was generated by randomly sampling without replacement a subset of reactions, corresponding to about one tenth of the total, from those available. The remainder of the reactions was used as query (or test) subset. In both subsets, the reactions could be uniquely identified as either positive or negative based on the raters’ classification. To increase the reliability of the results, the sampling procedure was performed once more generating a second pair of training and query subsets that were compared to the first pair and presented as supplementary material.

### MR method implementation

The MR algorithm was implemented as reported [15]. Briefly, an MR value for each reaction was calculated by identifying the maximum value of the series *R*_*n*_ *= (S*_*n*_*/S*_*n–1*_*)– 1* where *n* is a given cycle between the second and the last cycle of the reaction of amplification, *n–1* is the preceding cycle, *S* is the fluorescence emission and *R* is the ratio for the chosen cycle *n*. A five point moving average filter was applied to the ratio response, followed by cubic spline interpolation in order to obtain fractional cycle numbers (FCN) with a resolution of one hundredth of a cycle. For each reaction, the maximum of the *R*_*n*_ series (MR) provided the relative measure of reaction efficiency for the response and the correspondent FCN.

We introduced a filtering procedure to determine the reactive/non-reactive status of each reaction, which was implemented as follows. Using the training set, a histogram analysis was carried out with the Expectation and Maximization (EM) algorithm [24] implemented by the package *mixtools* for R [25]. This step produced an MR density distribution for both the positive and negative reactions and a series of cut-offs were generated to separate the means of the two populations. From this range, lower, upper and mid-point cut-offs could be established; the cut-off that minimised the stratification of samples between the raters’ classification and the MR output was chosen.

Since a common anomaly in qPCR are transients in the fluorescence signal during the early cycles of the PCR [15], MR values with a low FCN were expected to be higher than those in later cycle numbers. To handle this possible bias, a two cut-off strategy was implemented, using the lowest FCN of the positive reactions as a threshold to decide what cut-off to apply. For reactions with FCN below this point, the MR cut-off was based upon the maximum MR values recorded and multiplied by 1.5 to account for possible high responses in the query set. For reactions with an FCN above the lowest positive result, the cut-off was chosen with the described EM procedure. These cut-offs were then applied to the query set.

### Standard curves

A plasmid containing sequences of norovirus (NoV) genotype G.II (Genscript) was quantified at 1.48 × 10^10^ copies/μl. Serial dilutions were prepared in distilled water containing herring sperm carrier DNA. Serial 1:10 dilutions from 10^−5^ to 10^−9^ of the plasmid stock were prepared and measured in triplicates with the TAC assay. The standard curves were obtained by fitting linear regression models using the reciprocal of the dilution factor and either the Cq or FCN values; the slopes (*s*) of the models were used to calculate the efficiency of amplification (*E*) of the PCR using the formula: *E* = 10−^1/*s*^– 1 [26].

### Statistical analysis

All analyses were performed using R version 3.2.2. The inter-rater reliability was obtained by applying unweighted Cohen's kappa (κ) statistics and the agreement between more than two classifiers was determined by Fleiss' κ [27]. Cohen's and Fleiss' κ values were reported together with the 95% confidence interval and p-value, respectively. Slight, fair, moderate, substantial and almost perfect agreement rates were given for κ values ranging 0–0.20, 0.21–0.40, 0.41–0.60, 0.61–0.80 and 0.81–1, respectively [28]. Clinical accuracy, specificity, sensitivity, positive predictive value (PPV) and negative predictive value (NPV) for the CT and MR methods were calculated [29]. Median values were reported with the associated interquartile range.

## Results

### Classification of the clinical dataset

The clinical dataset was composed of 15 744 reactions obtained by testing 328 faecal specimens for a panel of 54 targets carried out over 50 TAC plates; 3456 wells of these plates did not contain clinical samples and therefore were not included in the present study. Overall, the agreement among the raters for issuing positive or negative results for this dataset was almost perfect (Fleiss' κ = 0.940, p-value<0.001). For the 269 reactions where the raters did not agree unanimously (1.71%), the Cq values showed a median of 36.5 (34.9–38.7) cycles and 89.2% were below 40 cycles. Using the consensus based on the raters’ classification, 1675 reactions resulted positive (10.6%). The CT analysis was performed with a set cut-off level of 0.2 florescence units whereas the baseline range was automatically selected by the *Viia7* software. Although the default range was defined between cycles 3 and 15, the actual values were between 1–3 and 3–44 cycles for the beginning and end of the baseline interval, respectively.

### Implementation of the MR method

**Fig 1:**
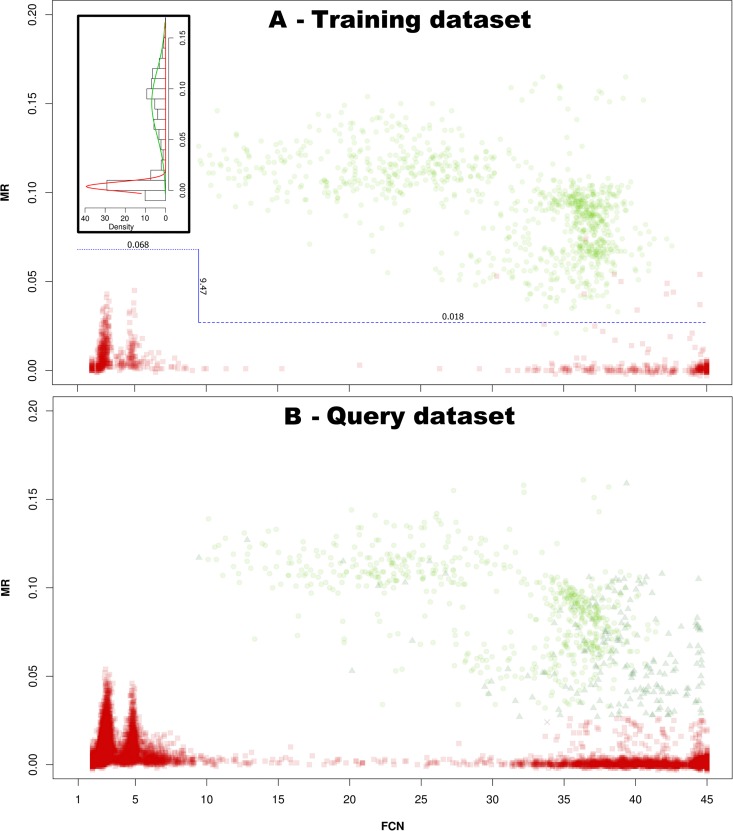
Clustering of TAC results in the FCN-MR plane. A: output of the MR method for the training dataset. Reactions were identified by a consensus classification as either positive (●) or negative (■) and are plotted in the FCN-MR plane, showing the different localization of the two populations. Since early cycles of amplification might be biased by transient in fluorescence that can generate high MR values, two MR cut-offs were applied to separate positive and negative results. For values below the lowest FCN of the positive population (9.47, solid line), 150% of the maximum MR observed in the region was used (0.068, dotted line). Histogram analysis was implemented to obtain MR values that could separate the positive and negative populations (insert). The mid-point between the lowest and highest of these separators was used to discriminate positive and negative results for reactions with a FCN values above the lowest FCN of the positive population (0.018, dashed line). B: the cut-off values empirically obtained by analysing the training dataset were applied to the query dataset in order to identify positive and negative reactions. The results are classifies as true positive (●), true negative (■), false positive (▲) and false negative (×) according to the consensus classification. The FCN axis has units defined in cycles whereas the MR axis is a-dimensional. The x-axis of the insert panel is given in MR values.

A subset of reactions was used to generate a training dataset obtained by randomly sampling without replacement 1920 reactions from the clinical dataset (12.2%); using the consensus value, the reactions could be unequivocally identified as either positive (n = 962) or negative (n = 958). The lowest FCN of the positive reactions within the training dataset was 9.47 cycles and the highest MR value for the reactions with FCNs below this cycle was 0.045 (Fig 1A). The cut-off for reactions of the query dataset with FCN values below 9.47 cycle was set at 1.5 × 0.045 = 0.068. For the reactions of the query dataset with FCN values above 9.47, three cut-offs were evaluated based on the results obtained by the histogram analysis: the upper (0.035), lower (0.018) and mid-point (0.027) of the provided range. Using the consensus classification as a guideline, the lower cut-off yielded 292 false positive reactions and no false negative calls when applied to the query dataset; on the other hand, the upper cut-off generated 160 false positive and 7 false negative results (data not shown). In order to minimize the amount of false positive and false negative results, the mid-point between these two cut-offs was chosen and applied to the query dataset.

### Classification of the query dataset

**Fig 2:**
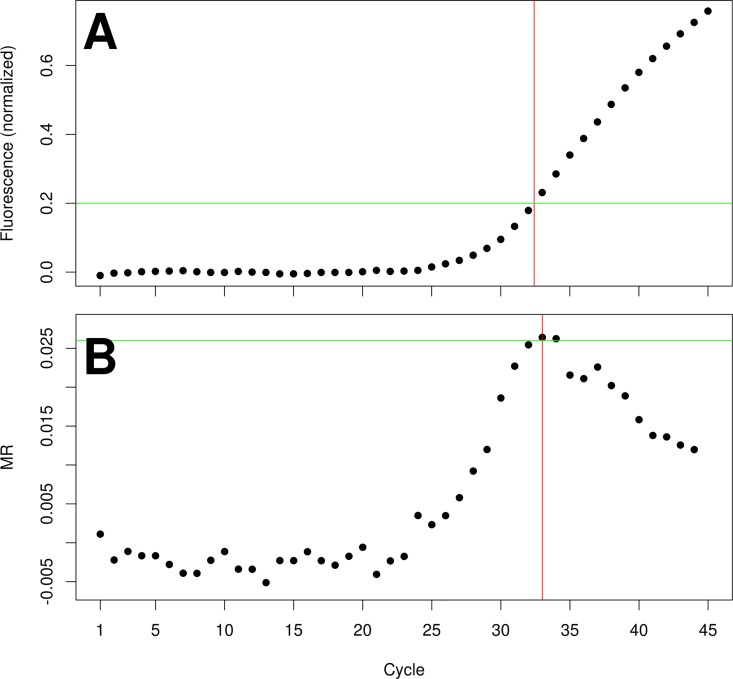
Representative amplification profile of the MR false positive results. Fluorescence amplification profile of the single reaction identified as false positive by the MR method in comparison to the consensus classification. A: the normalized fluorescence profiles indicates a proper amplification but the signal is weak, being only about three times the cut-off level of 0.2 fluorescence units (horizontal line). The Cq of this reaction (32.42 cycles) is reported (vertical line). B: the MR response for this reaction shows a good profile but the MR value (horizontal line) is below the empirical cut-off obtained using the training dataset, therefore the reaction was identified as negative. The FCN value (33.82 cycles) is depicted (vertical line).

The query dataset was composed of 13 824 reactions and was evaluated by the MR algorithm using the filtering parameters empirically determined (Fig 1B) and compared to the CT method (Table 1). The agreement between raters for the query dataset gave Fleiss' κ = 0.885 (p-value<0.001); the agreement between CT and MR methods in identifying positive and negative reactions gave Cohen's κ = 0.931 (0.919–0.944). The agreement between either CT and MR methods or the outcome provided by the raters gave Fleiss' κ of 0.884 and 0.902, respectively (p-value<0.001 in both instances). The stratification of the reactions by raters and analytical algorithms suggested that the MR method provided a higher proportion of true positive and negative results than the CT method. In particular, the MR algorithm provided 108 less false positive results than the CT method, according to the consensus classification; the median cycle value for the false positive reactions identified by the MR method was 36.16 (34.75–38.56). All the false positive reactions identified by the MR algorithm resulted also as false positive by the CT method. The MR method identified a single false negative reaction whereas the CT method did not produce false negative results; however the fluorescence profile of this reaction indicated that the signal was weak (Fig 2). The consensus-rated reactions showed that, in comparison to the CT method, the MR algorithm attained a slightly increased accuracy and specificity together with a better PPV, an equal NPV and a somewhat reduced sensitivity; this trend was also observed for the raters taken individually (Table 2). Only one positive reactions of the query set had a FCN below 9.47: the FCN was 9.45 and the MR was 0.117. There were 8218 negative reactions with a FCN below 9.47, whose maximum MR was 0.054; among these reactions, 13 resulted in a positive CT outcome with a median Cq of 41.61 (21.10–43.45) cycles.

**Table 1:** Stratification of reaction results by mathematical method and raters. The clinical dataset was subdivided in a training (n = 1920) and query (n = 13 824) subsets. The number of reactions identified as positive (+) or negative (–) by either the CT or the MR methods are clustered by the raters’ classification (A-C and a consensus generated by the median value between them).

		**Training dataset**							
		**Rater A**		**Rater B**		**Rater C**		**Consensus**	
		+	–	+	–	+	–	+	–
CT	+	976	4	956	24	945	35	944	36
	–	0	940	0	940	0	940	0	940
		**Query dataset**							
		**Rater A**		**Rater B**		**Rater C**		**Consensus**	
		+	–	+	–	+	–	+	–
CT	+	845	56	697	204	630	271	609	292
	–	0	12 923	1	12 922	0	12 923	0	12 923
MR	+	784	8	696	96	621	171	608	184
	–	61	12 971	2	13 030	9	13 023	1	13 031

**Table 2:** Comparison of the CT and MR methods. The assay parameters for the CT and MR methods are provided for each rater and for the consensus classification. PPV = positive predictive value; NPV = negative predictive value.

	Rater A		Rater B		Rater C		Consensus	
	CT	MR	CT	MR	CT	MR	CT	MR
Accuracy	0.996	0.995	0.985	0.993	0.980	0.987	0.979	0.987
Specificity	0.996	0.999	0.984	0.993	0.979	0.987	0.978	0.986
Sensitivity	1	0.928	0.999	0.997	1	0.986	1	0.998
PPV	0.938	0.990	0.774	0.879	0.699	0.784	0.676	0.768
NPV	1	0.995	1	1	1	0.999	1	1

A second pair of training (n = 1920) and query (n = 13 824) datasets was independently generated by randomly resampling the data of the available 15 744 reactions; the thresholds identified with the training set were applied to the query set. The results showed the same trend reported so far: namely, for the consensus classification, a slightly increased accuracy, specificity and Fleiss’ κ as well as a more substantial higher PPV of the MR method in comparison to the CT method (S1–S4 Tables).

### Reaction profiles

**Fig 3:**
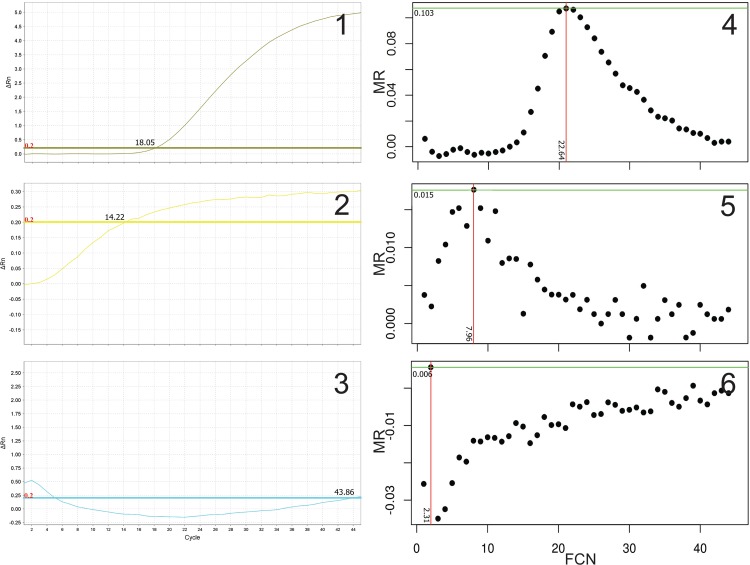
Examples of amplification profiles and MR responses. Representative amplification profiles of typical cases observed in this study. Sigmoid profile derived from a clear positive sample observed with the CT method (1) and the associated Gaussian profile of the MR response (4). Low fluorescence signal that crossed the threshold level giving a positive result with a low Cq value using the CT (2) but not with the MR (5) method. Transient peaks in fluorescence resulted in high Cq value with the CT method (3) but gave a negative result using the MR method (6). The y-axis is provided in different scales across the panels.

The false positivity of the CT method was typically due to slow raising or non-sigmoid fluorescence profiles that reached the cut-off level; remarkably these reactions did not produce a valid result using the MR method (Fig 3). Several reactions were characterized by a peak fluorescence in the early cycles followed by a slow but steady rise in fluorescence that in some cases reached again the threshold level at later cycles. These early peaks in fluorescence (transients) corresponded to two spikes of MR values between FCN 2–5 (Fig 1).

### Subset analysis

Since it is common practice with the CT method to remove the last cycles of amplification in order to reduce the false positive results, the MR method was applied to the query dataset once the reactions whose Cq was above 40 cycles (n = 72) were considered CT-negative. The results showed a slightly greater test accuracy and agreement with the consensus classification of the MR over the CT method (Table 3). In order to determine whether the results obtained thus far were not concealing a target specific bias, four subsets were considered: the external (bacteriophage MS2) and internal (bacterial 18S ribosomal RNA) controls of amplification, the NoV G.II and the *Campylobacter* species. The results showed that the MR algorithm provided agreement and accuracy equal or somewhat higher than the CT method.

**Table 3:** Agreement between mathematical methods and raters in sample subsets. The Fleiss' κ values obtained for selected subsets are stratified according to the raters one another or the raters together with either the CT or MR methods. The query subset was obtained by considering as negative the CT results with a Cq above 40 cycles; the other subsets included also Cq above this level to be consistent with the original clinical dataset. The p-values for the Fleiss' κ test were all lower than 0.001. The accuracy was based on the consensus classification. The number of reactions per group is reported (n).

	Query*	MS2	18S	NoV	*Camp*^*†*^.
n	13 824	130	131	253	195
κ (raters one another)	0.885	0.745	0.798	0.887	0.597
κ (CT method and raters)	0.901	0.796	0.787	0.907	0.580
κ (MR method and raters)	0.902	0.796	0.787	0.907	0.657
Accuracy (CT)	0.984	0.985	0.962	0.957	0.687
Accuracy (MR)	0.987	0.985	0.962	0.957	0.815

*Cq > 40 cycles were considered CT-negative.

^***†***^ *Campylobacter* species.

### Efficiency of amplification

**Fig 4:**
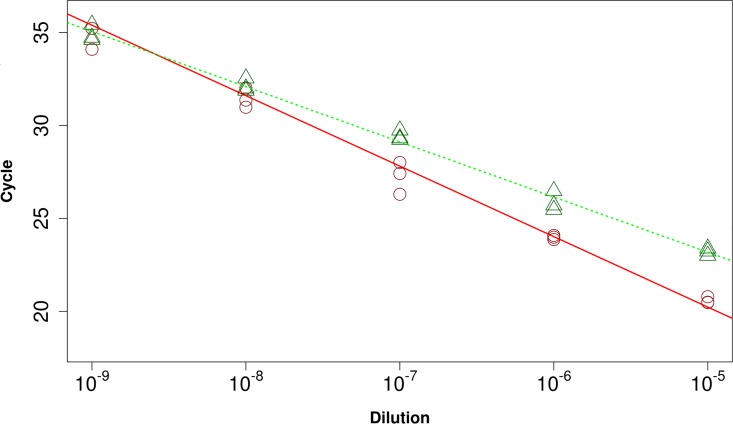
Standard curves obtained by CT and MR methods. The Cq (●) and FCN (▲) values for the different plasmid dilutions are depicted. The regression lines built on these values represent the standard curves for the CT (solid line) and MR (dotted line) methods.

The CT and MR methods were used to estimate the performances of the primer set targeting NoV G.II (Fig 4). The results showed that the CT method attained an efficiency of amplification of 0.836 compared to the 1.172 of the MR algorithm. The intercepts were 54.3 and 49.9 for the CT and MR methods, respectively. The coefficients of determination were 0.972 and 0.993 for the CT and MR methods, respectively. In particular, there were indications that the FCN values were more reproducible at the highest dilutions than the Cq counterparts.

## Discussion

The use of qPCR is widely implemented for both clinical and diagnostic purposes; in the latter case, the importance of providing results quickly and accurately is directly associated with a patient's well-being, thus these characteristics are paramount. Quantitative PCR platforms are evolving for higher throughput: for instance, Roche Diagnostic has developed a 1536 well format platform (*LightCycler 1536* Instrument) [30] and ThermoFisher Scientific is introducing a 3072 well format for the TAC assay (*OpenArray*) [31]; a PCR system allocating 300 000 simultaneous reactions has also been presented [32]. Since the validation of the qPCR data is typically done by visually inspecting the individual amplification profiles, the operators are facing an increasing labour burden and an enhanced risk of erroneously issuing clinical results. The evaluation of thousands of reactions is highly unlikely to be performed without a computerized aid, therefore reliable automated analytical tools for qPCR data analysis are required.

The widespread use of the CT method for the analysis of the qPCR data might imply differences between operators and laboratories due to (a) lack of consensus on the values to assign to the baseline range and threshold level, and (b) not account for possible reaction inhibition [5]. For instance, although multiple threshold levels are accepted in qPCR analysis to compensate for primer-specific idiosyncrasies [3], researchers and diagnostic operators tend to use a single value for all primer sets, thus ignoring possible sub-optimal amplification. Such bias can affect the precision of the qPCR assay: it has been estimated that differences of 2% in efficiency of amplification between reactions can lead to more than 400% discrepancy in target quantification [14].

Sub-optimal amplification were observed herein, in the form of transient peaks in fluorescence at the earliest cycles of amplification, despite the high standards of manufacturing and reproducibility that characterize the TAC assay. This behaviour has been previously reported in qPCR [15]; more specific studies in micro-fluidity might shed light on the causes of this phenomenon. In the present study, these early fluctuations often resulted in positive signals with the CT method whereas, in other instances, they tended to mask weak positive reactions.

The present study showed that the agreement of three independent raters in validating PCR results was almost perfect but not quite absolute; this difference was expected due to reactions with low template inputs. The raters that contributed to the present study indeed dealt differently with the reactions at hand. In particular, one rater classified as positive reactions with low amplification signal in order to increase the detection rate of the screening assay and rule-in as many cases as possible; the other raters, instead, identified as positive mostly the reactions with a sharper amplification signal. The MIQE guidelines for the analysis of the qPCR data highlight reactions with quantitative cycles above 40 as indicative of poor amplification but there is no clear explanation whether such results should be discarded altogether, accepted as weak positive or repeated [1]. In the present study only one tenth of the results where the raters did not unanimously agree had a Cq above 40 cycles, suggesting that the issue of subjectivity in clinical reporting cannot be solved with an arbitrary cut-off.

The use of assumption-free methods in the analysis of qPCR data would grant the standardization of the validation methods and provide objective results. Among the assumption-free methods available, we have chosen the MR algorithm because of its simple working principle that does not require regression fitting, its capability to clearly discriminate positive from negative reactions [15] and because it could be conveniently implemented in the cross-platform R environment. The MR algorithm might easily be translated into other interpreted or multi-purpose languages such as Python, MATLAB and Java to attain a completely stand-alone and portable application that can assist scientists in issuing qPCR results, in particular in high throughput laboratories. One issue of the MR method is the requirement for the amplification fluorescence values to be positive in order to avoid division by zero errors.

We have improved the MR algorithm by introducing a filtering procedure based on empirical cut-offs of the MR parameter. Due to transients in the early cycles of amplification, we employed two cut-offs and used the lower FCN of the positive samples of a training set as a watershed for their application. In the early cycles, the maximum MR observed for the negative results was used; this value was incremented by a given number to take in account the possible presence of negatives with high MR in the query set. We chose a multiplier of 1.5 in analogy to the one used to identify the moderate outliers in the box-and-whiskers plot [33]. Remarkably, the negatives reactions with low FCN identified in the query dataset had MR values well below this point, indicating that the multiplier could be adjusted even further without affecting the robustness of the algorithm. For reactions with high FCN values, the MR cut-off was obtained by an assumption-free approach (EM algorithm) based on the localization of positive and negative reactions of the training dataset in the FCN-MR space.

The training dataset could be updated along with the application of the qPCR assay, increasing the effectiveness of the parameters required by the MR filtering algorithm. The information provided by the training dataset could be used as a whole or subdivided in individual targets according to the specific requirements. In the present study, we chose to consider all the different primer sets as a single group, an approach that made it difficult to identify MR cut-off values that could fit the specific fluorescence pattern of the different primers sets. Emphasis was given herein to optimise the robustness of detecting positive samples, a goal achieved by the MR algorithm with slightly higher specificity and κ values, NPV close to 100% and, more importantly, PPV substantially increased in comparison to the CT method. These features are essential to evaluate the effectiveness of a diagnostic assay [29,34]. The EM parameters used to filter the MR results can be adjusted to better fit the requirements of the individual laboratories, including the proportion of false positive results, assuring that the results could be shaped according to the actual amplification data.

We also performed an assessment of the MR method for quantitative purposes. The data gathered showed that the CT and MR methods provided an efficiency of amplification slightly below and above, respectively, the suggested range of optimal efficiency of amplification of 90–110 percent [35]. The lower CT efficiency might result in underestimation of the amount of target with repercussions, for instance, in drug therapy management. It has been previously noted that the CT and MR methods might under- and over-estimate, respectively, quantitative cycles in suppressed responses [15]; further work could assess whether this divergence holds also for non-inhibited reaction.

The present study had some limitations. First, the overall strategy was for screening purposes with no sample replication; nevertheless, the limited data gathered herein from plasmids replicates suggested that the MR method had the potentialities for more reproducible calculation of quantitative cycles than the CT method. Second, the vast majority of the samples were negative, therefore there was a loss in amplification data; however the high number of reactions analysed herein allowed for a large enough sample set to draw conclusions on the qPCR analytical methods. Third, the dataset was generated from a heterogeneous number of targets rather than a single primer set, thus some of the amplification differences between them might have introduced a bias in the data analysis. We addressed such possibly by analysing subsets of the available data; the results suggested that the MR algorithm is at least as accurate as the CT method regardless of the primer set. Fourth, this was a project with very limited resources, therefore only three raters could be recruited to assess a small fraction of the qPCR data produced by a single laboratory of molecular diagnostic. Bigger research implementations could provide a more thorough understanding of the variability between operators and laboratories in issuing qPCR data. In addition, the small number of raters might have introduced a bias in the results. Fifth, we assumed that the clinical dataset was large enough to contain independent data and we used a simple random sampling method to generate the training and query subsets. This approach is not optimal and might have introduced a bias in the results. A more appropriate method of resampling is the *k*-fold cross validation, where a dataset with *n* data is subdivided in *k* subsets of *n/k* values each used in turn as query dataset [36]. Our limited resources made difficult to apply a cross validation approach to our dataset, but we address the sampling problem by analysing a second dataset generated independently from the first one; the results were identical to those observed in the first dataset, strengthening our claims.

The work carried out herein showed interesting outcomes. There is a number of reports related to the inter-rater agreement for diagnostic assays, particularly involving visual scoring [37–39], however those including PCR data are mostly focused at comparing this assay with other tests rather than the variability of PCR results issued by multiple operators [40]. To the best of our knowledge, this is the first study assessing the agreement between operators for the qPCR assay. Other studies based on the development of qPCR method indicated the comparison to CT in terms of template quantification, efficiency of amplification and quantitative cycles but did not report the inter-rater agreement or the accuracy of the assay [7–14,41]. The present study was also the first to apply the MR method to the analysis of TAC data. Furthermore, the use of the TAC assay allowed the evaluation of thousands of reactions in a highly reproducible fashion, providing a good overlook of the behaviour of the reactions profiles.

## Conclusions

In conclusion, the present study demonstrated the potentialities of the improved MR method for the discrimination between positive and negative qPCR results in an assumption-free fashion. In addition, the MR method displayed capabilities for a reproducible determination of the quantitative cycles that can be used for the assessment of standard curves and template copy calculation. Finally, the present study showed that computer-assisted data evaluation could speed-up the analysis, identify aberrant reactions that might be missed by the human eye, and reduce the risk of result issuing errors.

## Supporting Information

S1 File Working example of the filtered *maxRatio* algorithm introduced in the present work. The compressed folder contains a vignette explaining the procedure (‘S1 vignette.pdf’), an executable (‘S1.R’), three files with qPCR data (‘plate 1.txt’, ‘plate 2.txt’ and ‘plate 3.txt’) and a table containing the classification of the reactions provided by the raters (‘rating table.txt’). The S1.R executable produces two files as output: ‘training_file.txt’ and ‘results_file.txt’. In order to work, S1.R requires the package ‘mixtools’ (https://cran.r-project.org/web/packages/mixtools/index.html) to be loaded in the R environment.
(ZIP)

S1 Table Stratification of reactions for the second set of data. The clinical dataset was subdivided in a training (n = 1920) and query (n = 13 824) subsets and was generated independently from the datasets reported in the main text. The number of reactions identified as positive (+) or negative (–) by either the CT or the MR methods are clustered by the raters’ classification (A-C and a consensus generated by the median value between them).
(DOCX)

S2 Table Comparison of the CT and MR methods for the second set of data. The assay parameters for the CT and MR methods are provided for each rater and for the consensus classification. PPV = positive predictive value; NPV = negative predictive value.
(DOCX)

S3 Table Agreement between mathematical methods and raters for the second set of data. The Fleiss' κ values obtained for query datasets are stratified according to the raters one another or the raters together with either the CT or MR methods. The p-values for the Fleiss' κ test were all lower than 0.001.
(DOCX)

S4 Table Parameters obtained from the second training dataset. The values reported herein were used to set the filtering parameters of the training dataset.
(DOCX)
