# Supplementary material for: Analysis of TaqMan Array Cards Data by an Assumption-Free Improvement of the maxRatio Algorithm Is More Accurate than the Cycle-Threshold Method
Source: PLoS One. 2016 Nov 9;11(11):e0165282. doi: 10.1371/journal.pone.0165282 (PMC5102466; doi:10.1371/journal.pone.0165282)
Supplement: S1 File — The compressed folder contains a vignette explaining the procedure (‘S1 vignette.pdf’), an executable (‘S1.R’), three files with qPCR data (‘plate 1.txt’, ‘plate 2.txt’ and ‘plate 3.txt’) and a table containing the classification of the reactions provided by the raters (‘rating table.txt’). The S1.R executable produces two files as output: ‘training_file.txt’ and ‘results_file.txt’. In order to work, S1.R requires the package ‘mixtools’ (https://cran.r-project.org/web/packages/mixtools/index.html) to be loaded in the R environment. (ZIP) [file pone.0165282.s001.zip › S1 vignette.pdf]

Vignette for the use of the *maxRatio* function implemented in the supplementary material S1.

The supplementary material S1 contains a script for the R environment and the trial files; the latter consist of qPCR data generated by the *Viia7* thermal cycler (Thermo Fisher Scientific Instruments). The S1.R script is simply a working function to analyse exemplification data for the use of the MR algorithm; it is a crude but functional script and better implementations could be obtained. Nevertheless, it can be applied to any data provided by the user, granted that the structure of the data follows the indications reported herein.
The *maxRatio* is a ratio between raw fluorescence values collected at two consecutive cycles, therefore it is an assumption-free (since it does not require any input from the operator such as the choice of the threshold) and a-dimensional value that accounts for the specific characteristics of the amplification data. The *maxRatio* algorithm returns two values: MR (the actual ratio) and FCN (the cycle number associated to MR); these values need to be filtered out in order to discriminate between positive and negative reactions. This filtering step is obtained by the statistical separation of reactions known to be either positive or negative; in this implementation we have used the expectation (E) and maximization (M) algorithm.
The following notes are aimed at increasing the understanding of the structure and application of the S1.R script. A working example is also provided.

Notes.

1. The S1.R script defines the following working functions:
   i. 'maxPrior', to analyse the qPCR data of a training dataset
   ii. 'maxParms', to determine the best filtering parameters for the results obtained by 'maxPrior'
   iii. 'maxRatio', to analyse the qPCR data of a query dataset
2. These functions have different dependencies:
   i. 'maxRatio' and 'maxPrior' do not require dependencies
   ii. 'maxParms' requires the library "mixtools" to execute the EM algorithm in order to identify the threshold needed to discriminate positive and negative reactions
3. Both 'maxRatio' and 'maxPrior' calculate MR and FCN values using the subfunction 'maxCalc'.
4. Data is loaded into the R environment using the subfunction 'maxLoad'.
5. The 'maxPrior' function accepts the following input:
   i. a flatfile (.txt) in tab-delimited format with the following headings:
      a) " " = unique row number of the records for dataframe conversion
      b) "Well" = qPCR well number
      c) "Cycle" = qPCR cycle number
      d) "Target.Name" = qPCR primer set name
      e) "Rn" = raw fluorescence reading
   ii. a flatfile (.txt) in tab-delimited format with the following headings:
      a) " " = unique row number of the records for dataframe conversion
      b) "Well" = qPCR well number refering to the same well as the other file
      c) "outc" = dummy variable taking 1 for positive reactions
6. The output of the 'maxPrior' function is a table provided in a tab-delimited flat file ("training_file.txt") containing the following fields:
   i. " " = unique row number of the records for dataframe conversion
   ii. "well" = qPCR well number
   iii. "fcn" = FCN value
   iv. "mr" = MR value
   v. "outc" = ratification provided by the operator
7. The 'maxParms' functions accepts as input "training_file.txt".
8. The output of the 'maxParms' function is a list of parameters provided on screen with the following fields:
   i. "Turning FCN" = tcycle
   ii. "Threshold < turning FCN" = thresh1
   iii. "Threshold >= turning FCN (lower)" = thresh2
   iv. "Threshold >= turning FCN (mid-point)" = thresh2
   v. "Threshold >= turning FCN (higher)" = thresh2
9. These parameters are fed manually into the 'maxRatio' function.
10. The "thresh2" parameter is chosen among the lower, mid-point and high values provided by

'maxParms'.

11. The 'maxRatio' function accepts the following input:
    i. a flatfile (.txt) in tab-delimited format with the following headings:
        a) " " = unique row number of the records for dataframe conversion
        b) "Well" = qPCR well number
        c) "Cycle" = qPCR cycle number
        d) "Target.Name" = qPCR primer set name
        e) "Rn" = raw fluorescence reading
    ii. the numeric value of the threshold ("thresh1") for the reactions with a FCN below the lowest FCN of the positive reactions of the training set
    iii. the numeric value of the threshold ("thresh2") for the reactions with a FCN above the lowest FCN of the positive reactions of the training set
    iv. the numeric value of the FCN ("tcycle") of the positive reactions of the training set
12. The output of the 'maxRatio' function is a table provided in a tab-delimited flat file ("results_file.txt") containing the following fields:
    i. "Well" = qPCR well number
    ii. "Target" = qPCR primer set name
    iii. "FCN" = FCN value
    iv. "MR" = MR value
    v. "Width" = distance between ½MR and FCN
    vi. "Result" = dummy variable taking 1 for positive results of the MR algorithm

Example.
In the following example all files are assumed to be present in the working folder; alternatively a path could be provided to point to the location of the needed files or the native function `setwd` could be used to determine the required working folder.
In addition, the package mixtools is required for the filtering step; this can be installed with the command:

```
> install.packages("mixtools")
```

And then loaded in the R environment with the command:

```
> library(mixtools)
```

The S1.R script is loaded into the R environment using the native function `source`:

```
> source("S1.R")
```

Let's assume that the amplification data for the training set is provided in the file "plate 1.txt", which was obtained by exporting such data using the *Viia7* software from a TAC run. The "Amplification data" spreadsheet generated by the Viia7 software was then rearranged by removing the initial lines (1-29) containing the detail of the qPCR run and adding an empty column with a progressive number. Such rearrangement was performed herein with LibreOffice *Calc*. The rating of the different reactions contained in "plate 1.txt" as either positive or negative are determined manually by trained operators and provided in a separate file named "rating table.txt".
The files "plate 1.txt" and "rating table.txt" are then fed to the `maxPrior` function as follows:

```
> maxPrior("plate 1.txt", "rating table.txt")
Results written in 'training_file.txt'
```

The subsequent step is to feed the "training_file.txt" to the `maxParms` function in order to obtain the values for "tcycle", "thresh1" and "thresh1":

```
> maxParms("training_file.txt")
mixtools package, version 1.0.4, Released 2016-01-11
This package is based upon work supported by the National Science Foundation
under Grant No. SES-0518772.

number of iterations= 28
                                      Value
Turning FCN                          11.860
Threshold < turning FCN               0.063
Threshold >= turning FCN (lower)      0.007
Threshold >= turning FCN (mid-point)  0.036
Threshold >= turning FCN (higher)     0.064
```

Let's now assume that the query data is contained in the files “plate 2.txt” and “plate 3.txt”, which have been arranged in the same way as “plate 1.txt”. The function `maxRatio` can then be called and the results saved into the file “results_file.txt”. This file is first initialized to provide the headers of the columns; the data is then appended to it. The procedure can be implemented with the following commands that:

```
> result.data <- data.frame(Well = numeric(), Target = character(), FCN =
numeric(), MR = numeric(), Width = numeric(), Result = numeric())
> write.table(result.data, file = "results_file.txt", sep="\t", quote = FALSE,
row.names = FALSE, col.names = TRUE, append = FALSE)
> for (i in 2:3) {
result.data <- maxRatio(paste("plate ", i, ".txt", sep=""), 0.063, 0.036,
11.860)
write.table(result.data, file = "results_file.txt", sep="\t", quote = FALSE,
row.names = FALSE, col.names = FALSE, append = TRUE)
  }
```

Now “results_file.txt” contains a table with all the samples analysed, classified as either positive or negative based on the MR result through the “Result” field. The first 384 rows correspond to the “plate 2.txt” file and the rest to “plate 3.txt”.
