# Supplementary material for: Analysis of TaqMan Array Cards Data by an Assumption-Free Improvement of the maxRatio Algorithm Is More Accurate than the Cycle-Threshold Method
Source: PLoS One. 2016 Nov 9;11(11):e0165282. doi: 10.1371/journal.pone.0165282 (PMC5102466; doi:10.1371/journal.pone.0165282)
Supplement: S1 Table — The clinical dataset was subdivided in a training (n = 1920) and query (n = 13 824) subsets and was generated independently from the datasets reported in the main text. The number of reactions identified as positive (+) or negative (–) by either the CT or the MR methods are clustered by the raters’ classification (A-C and a consensus generated by the median value between them). (DOCX) [file pone.0165282.s002.docx]

|  |  | **Training dataset** | | | | | | | | | | | | | | | |
| --- | --- | --- | --- | --- | --- | --- | --- | --- | --- | --- | --- | --- | --- | --- | --- | --- | --- |
|  |  | **Rater A** | | | | **Rater B** | | | | **Rater C** | | | | **Consensus** | | | |
|  |  | + | | – | | + | | – | | + | | – | | + | | – | |
| CT | + | 976 |  | 6 |  | 959 |  | 23 |  | 945 |  | 37 |  | 960 |  | 22 |  |
|  | – | 0 |  | 938 |  | 0 |  | 938 |  | 0 |  | 938 |  | 0 |  | 938 |  |
|  |  | **Query dataset** | | | | | | | | | | | | | | | |
|  |  | **Rater A** | | | | **Rater B** | | | | **Rater C** | | | | **Consensus** | | | |
|  |  | + | | – | | + | | – | | + | | – | | + | | – | |
| CT | + | 845 |  | 54 |  | 694 |  | 205 |  | 630 |  | 269 |  | 715 |  | 184 |  |
|  | – | 0 |  | 12 925 |  | 1 |  | 12 924 |  | 0 |  | 12 925 |  | 0 |  | 12 925 |  |
| MR | + | 820 |  | 23 |  | 694 |  | 149 |  | 630 |  | 213 |  | 715 |  | 128 |  |
|  | – | 25 |  | 12 956 |  | 1 |  | 12 980 |  | 0 |  | 12 981 |  | 0 |  | 12 981 |  |
