# Supplementary material for: Analysis of TaqMan Array Cards Data by an Assumption-Free Improvement of the maxRatio Algorithm Is More Accurate than the Cycle-Threshold Method
Source: PLoS One. 2016 Nov 9;11(11):e0165282. doi: 10.1371/journal.pone.0165282 (PMC5102466; doi:10.1371/journal.pone.0165282)
Supplement: S2 Table — The assay parameters for the CT and MR methods are provided for each rater and for the consensus classification. PPV = positive predictive value; NPV = negative predictive value. (DOCX) [file pone.0165282.s003.docx]

|  | **Rater A** | | **Rater B** | | **Rater C** | | **Consensus** | |
| --- | --- | --- | --- | --- | --- | --- | --- | --- |
|  | CT | MR | CT | MR | CT | MR | CT | MR |
| Accuracy | 0.996 | 0.997 | 0.985 | 0.989 | 0.981 | 0.985 | 0.987 | 0.991 |
| Specificity | 0.996 | 0.998 | 0.984 | 0.989 | 0.980 | 0.984 | 0.986 | 0.990 |
| Sensitivity | 1 | 0.970 | 0.999 | 0.999 | 1 | 1 | 1 | 1 |
| PPV | 0.940 | 0.973 | 0.772 | 0.823 | 0.701 | 0.747 | 0.795 | 0.848 |
| NPV | 1 | 0.998 | 1 | 1 | 1 | 1 | 1 | 1 |
