# Supplementary material for: Analysis of TaqMan Array Cards Data by an Assumption-Free Improvement of the maxRatio Algorithm Is More Accurate than the Cycle-Threshold Method
Source: PLoS One. 2016 Nov 9;11(11):e0165282. doi: 10.1371/journal.pone.0165282 (PMC5102466; doi:10.1371/journal.pone.0165282)
Supplement: S3 Table — The Fleiss' κ values obtained for query datasets are stratified according to the raters one another or the raters together with either the CT or MR methods. The p-values for the Fleiss' κ test were all lower than 0.001. (DOCX) [file pone.0165282.s004.docx]

| **Group** | **Value** |
| --- | --- |
| Raters one another | 0.885 |
| CT method and raters | 0.885 |
| MR method and raters | 0.896 |
