# Supplementary material for: Analysis of TaqMan Array Cards Data by an Assumption-Free Improvement of the maxRatio Algorithm Is More Accurate than the Cycle-Threshold Method
Source: PLoS One. 2016 Nov 9;11(11):e0165282. doi: 10.1371/journal.pone.0165282 (PMC5102466; doi:10.1371/journal.pone.0165282)
Supplement: S4 Table — The values reported herein were used to set the filtering parameters of the training dataset. (DOCX) [file pone.0165282.s005.docx]

| **Parameter** | **Value** |
| --- | --- |
| Lowest FCN for positive samples (FCN+) | 6.34 |
| Threshold below the lowest FCN+ | 0.078 |
| Threshold above the lowest FCN+ | 0.018 |
